# Epidermolysis Bullosa: Practical Clinical Tips From the Field

**DOI:** 10.7759/cureus.53774

**Published:** 2024-02-07

**Authors:** Aaron Tabor, Jo Ann K LeQuang, Joseph Pergolizzi

**Affiliations:** 1 Research, No Baby Blisters, Colorado Springs, USA; 2 Scientific Communications, NEMA Research, Inc., Naples, USA; 3 Pain, NEMA Research, Inc., Naples, USA

**Keywords:** baby blister, rare skin disease, dystrophic epidermolysis bullosa, recessive dystrophic epidermolysis bullosa (rdeb), epidermolysis bullosa simplex, junctional epidermolysis bullosa, epidermolysis bullosa

## Abstract

Epidermolysis bullosa (EB) is a rare genetic condition characterized by fragile skin caused by impaired adhesion between the dermis and epidermis. EB is present at or near birth. There is no cure and treatments are supportive. Children with EB are at elevated risk of squamous cell cancer. Under ideal circumstances, EB patients benefit from interdisciplinary care teams who can offer state-of-the-art treatments. In reality and particularly in less-developed nations, care can be limited. In all cases, families dealing with a member with EB face great challenges in caregiving, much of which is managed at home, and incur great financial expenses for dressings, equipment, transportation, and out-of-pocket expenses. While research groups are working to find a cure for EB, clinicians working with EB patients around the world have found practical and relatively inexpensive tips to make life easier for people with EB. NoBabyBlisters.org, a nonprofit organization actively supplying monthly medical supplies for EB children on five continents and working on EB research, has innovated, developed, collected, and now offers here seven such practical and actionable items learned from its experience in the real world assisting children in less-developed nations, typically with hot climates. These are based on real-world clinical experience dealing with a complex disorder under challenging circumstances. The goal of this short paper is to provide advice to EB caregivers and their loved ones that may make things easier and enhance quality of life, including blister and pain reduction.

## Introduction and background

The rare genetic disorder epidermolysis bullosa (EB), a genodermatoses characterized by skin fragility, presents in different forms with different prognoses and courses of supportive treatment [1]. The four main types of EB are EB simplex, dystrophic EB, junctional EB, and Kindler syndrome, but more than 30 subtypes have been recognized within those four main types, based mainly on the plane of cleavage within the skin [2]. EB occurs due to genetic mutations, with certain types autosomal dominant and others autosomal recessive. The primary manifestation may be described as impaired adhesion between dermis and epidermis, resulting in blisters and mucocutaneous fragility [2], but manifestations may be mild, severe, or even life-threatening and may affect eyes, ears, airways, and gastrointestinal systems [3].

The optimal treatment for this incurable condition is a multidisciplinary supportive palliation, aimed at reducing the risk and severity of blistering, thorough wound care, and symptomatic relief. When possible, efforts should be made to promote patient comfort, well-being, and quality of life. People with EB are at elevated risk for potentially fatal squamous cell carcinoma (SCC) [2]. People with severe recessive dystrophic EB are often affected by early and aggressive forms of SCC [4].

Comprehensive epidemiologic data on EB are sparse, but certain nations have published recent epidemiologic data. In England and Wales, there were 2,361 people with EB with a prevalence of 34.8 per million for all EB types. In this region, 1200 babies with EB arrived in the past 20 years (from 2002 to 2022) [5]. The American National EB Registry reports much lower numbers, with an estimated prevalence of 8.2 per million live births [6]. Slovenia reports 20 per million [7]. A population-based study in Germany found the incidence was 45 per million live births [8]. A Dutch registry reported 41.3 per million live births [9]. In Iran, there were 538 EB patients reported in 2021, or 6.72 patients per 100,000 persons [10]. Global EB prevalence has been estimated at 1 per 18,000 live births [11]. The variations in numbers may be the result of difficulties in diagnoses, small sample sizes, greater awareness of EB in some cases, and better and more frequent genetic testing in certain populations.

Since EB has no cure, treatments are palliative. There are few dedicated clinics or specialists for this condition, and many caregivers must work out their own remedies and systems at home with little guidance or support. The aim of this paper is to present a series of practical, actionable things that caregivers can do for people with EB to reduce blisters, pain, and skin infections. These are simple steps, some requiring very little money, and they may provide comfort, relief, and enhanced quality of life to the person with EB. All of these tips or “hacks” were developed, funded, implemented, and practiced among NoBabyBlisters.org doctors and staff working for EB patients on behalf of the charity No Baby Blisters. They have not been tested in clinical trials or deployed in a scientific way, but they have been put into practice in the care of children with this rare disease. This is not a comprehensive literature review but rather a report of practical tips from clinical work with people with EB and their caregivers.

## Review

EB treatment is supportive and aimed at relieving symptoms which can sometimes be debilitating. Efforts must be made to protect the skin and promote healing while keeping the person with EB as comfortable and resistant to infection as possible. Since EB can place a tremendous burden on the family, psychosocial counseling as well as supplemental services may be needed for the patient, caregiver(s), and the family [3]. Healthcare resources dedicated to EB are limited, even in the wealthiest nations, and developing nations may offer no services for EB. EB is best treated with an interdisciplinary care team, but few clinicians have experience with managing the many aspects of the disease. Many EB babies, in both rich and poor nations, depend on home care by overtaxed family members who get little information or help from the local healthcare services.

The costs of care for EB patients are high. In addition to the burden on unpaid caregivers and family stress, financial costs can be overwhelming. A survey conducted of 204 EB patents from eight European Union nations estimated annual direct costs per person per year to range from 9,509 to 49,233 Euros (approximately US$10,193 to US$52,777). These surveyed costs varied by type of EB, disability level, and age and did not include indirect costs [11]. Indirect costs, such as informal care and hidden “social costs,” such as lost productivity or reduced quality of life, are difficult to quantify [12]. The dressings required for wound care can cost from US$11 to US$128 per day for a neonate and are higher for older patients or those with larger and more complex wounds [13]. A survey in France of 77 parents raising EB children (mean age 7.5 years) found all respondents had out-of-pocket expenses not reimbursed by the healthcare system; the mean for these expenses was 4,129 ± 4,321 Euros per year (US$4,418 ± US$4,623). Despite these efforts and expenditures, the health-related quality of life remains low in the EB population [11].

Caring for a person with EB requires nearly full-time attention, which can take one or more persons in the household away from a paying job [14]. People with EB are often profoundly disabled and may experience difficulties with ambulation, toileting, pain control, and psychological distress. All of these conditions can be extremely taxing on a household [15] and this is compounded by the fact that local healthcare services offer few answers and little practical support. The rarity of this condition means that families with an EB child do not necessarily have ready contact with others in a similar situation for help, advice, and support.

**Figure 1:**
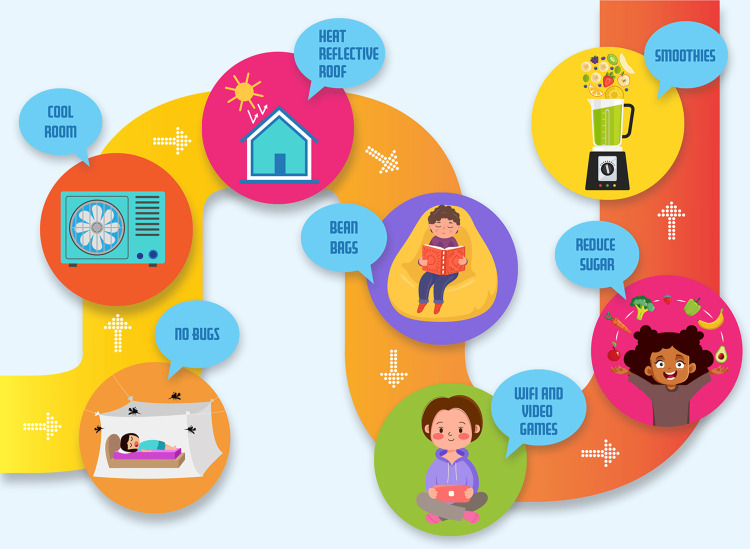
Simple tactics that can help minimize the suffering of EB children from real-world clinical experience Credit: Original art from No Baby Blisters by Belinda Kinkade of B-Creative. EB: Epidermolysis bullosa

No Baby Blisters is a charitable organization that works with EB patients and their families and is funding monthly medical supplies and research into finding curative approaches to EB. In this global work, field personnel, clinicians, executives, EB patients, and their families have learned and shared some practical strategies to help manage this condition. These are not advanced medical concepts but simple and tested strategies that can work in harsh, real-world environments (see Figure 1).

Housing security: get rid of bugs

Insect bites and stings can cause great distress to delicate skin. Systemic allergic reactions to insect bites occur in less than 1% of children and approximately 3% of adults but are of particular concern for the EB population [16,17]. Children are less likely than adults to have anaphylaxis following insect bites but are more likely to develop cutaneous symptoms which can be devastating for children with EB [18]. EB is associated with pruritus as well, which can be exacerbated by bug bites.

If there are holes or loose areas in walls, floors, or roofs that might permit insects to enter, these should be repaired or patched. Small cracks barely visible to the eye can be sufficient to allow bugs to enter. Sealant may be helpful. Even brick houses are susceptible to this sort of problem because insects can enter through weep holes or gaps in brick veneer. A good plan is to walk around the exterior of the house slowly to identify gaps, holes, and entry points. Bushes, plants, or tall grass that grows up close to the house should be trimmed back since this sort of foliage can provide an easy “bridge” for insects to get to the house.

Inside the house, extreme cleanliness and frequent sweeping or vacuuming can keep insects at bay. Remove cobwebs if they build up. Store food in sealed containers or otherwise away from bugs.

Mosquito intrusion can be prevented with screens over the window and mosquito netting over the bed for nighttime when mosquitoes are most active. Attention is needed to keep screens in good repair and to patch holes and tears. Fleas are also most active at night and can be deterred by frequent washing of bed linens. Simple flea traps using light and a sticky pad or water can be helpful. Ants, spiders, and other insects may climb up bed frames to get to the bed, so placing the legs of the bed in cups of water limits Hymenoptera and arachnids, because ants and spiders would be unable or highly unlikely to navigate this sort of obstacle.

When possible, the charity often pays to make obvious home repairs, such as closing holes or patching walls. These fixes need not be major repairs; it often suffices to do basic stop-gap measures.

In addition to reducing insects in the house, these methods may reduce the fears of the person with EB. An insect bite can be excruciatingly painful, so living in a home where insect bites are common can create an extremely unpleasant even panicky sensation. These steps may not prevent all insects from coming into the house, but reducing insects and making it a mission of the house to keep insects out can be reassuring to the person with EB.

Cool down with air conditioning

People with EB suffer less when it is cool compared to when it is warm. Climate control in the form of air conditioning can provide a great deal of soothing relief to people with EB. To reduce expenses, a small air conditioner can be set up in a single room that is closed off with a door or even with plastic sheeting to create a “cool zone” of comfort for the person with EB. If this is not possible or if rolling brownouts mean that cooling can be interrupted without notice, having paper fans and spray bottles to mist water near the person with EB can be used for a quick cool-down. When possible, keep the “cool space” small and separate from the rest of the living quarters so that minimal cooling can have a maximum effect.

In some instances, the charity has been able to build a small room with real walls that can be air-conditioned to a low temperature. This creates a chilled “safe room” in the house for an EB child.

Heat-reflective roofs

Dark roofs, particularly dark metal roofs, act to absorb sunlight and can transfer some of that absorbed heat to the house. A so-called “cool roof” reflects sunlight so that it bounces off the house and keeps the house cooler [19]. While there are elaborate cool roofing systems, an easy way to get a do-it-yourself cool roof is to paint the roof with reflective paint. Reflective paints are available for painting wooden, concrete, or metal surfaces. The use of a cool roof of any type can lower the temperature inside the house, benefiting the person with EB. Compared to conventional roofs, a cool roof can lower the roof temperature by as much as 50 degrees Fahrenheit [20]. The charity has installed several of these roofs in houses in hot sunny climates and has amassed early positive experience with this technology.

Bean bag chairs

When skin is very fragile, unyielding surfaces that look like wooden or metal chairs, can tear or harm the delicate skin. Wooden or metal furniture also forces the weight to be concentrated on a few pressure points, which may be susceptible to damage. When the charity first began working with individual families, it was observed that EB children spent significant time sitting on bamboo mats, hard clay, or metal materials. Bean bag furniture, such as bean bag chairs or sofas, can be far more comfortable for a person with EB. The stuffing in the furniture distributes the pressure over much wider areas of the body, reducing pressure on limited areas [21]. Bean bag chairs also allow the child to recline, which they may find more comfortable for reading, doing school work, or using mobile phones or video game devices. Since the materials yield to the child’s form and posture, bean bags can accommodate their specific needs without damaging the skin.

Video games and good WiFi

It has been the charity’s experience that EB children, like most other children, love to use tablets and electronic devices. When these children were given access to video games or other on-screen entertainment, they quickly adapted to them and used them frequently. This is beneficial to them because video games and other screen-based forms of distraction are currently being evaluated as ways to relieve anxiety, reduce fear, and even control pain [22]. Children in particular are very amenable to this form of distraction [23]. The charity found that EB children eagerly started using these devices, including for school work. This requires an internet connection with WiFi and a device (phone, apps, game console, tablet). The charity has often provided these tools to patients as ways to keep the EB child engaged, entertained, learning, and distracted from their painful condition.

Manage junk food and reduce sugar

Sugar and sugary beverages are associated with numerous health conditions, including low-grade chronic inflammation [24]. Further, sugary foods have been associated with neuroadaptations such that eating sugar can cause one to crave more sugar, forming a vicious cycle [25,26]. While sweet treats can be pleasurable, particularly for EB children who have so few outlets for enjoyment, they can increase inflammation which, in turn, can increase pain and discomfort levels. Moreover, high intake of sugar has also been associated with weight gain and cardiometabolic disease [27]. The recent PREDIMED (Prevención con Dieta Mediterránea (Prevention with Mediterranean Diet)) trial has found that the consumption of simple sugar in soft drinks and juices increased the overall rate of cancer, cancer mortality, and all-cause mortality [28].

Blenders for smoothies

People with EB can experience mucosal involvement, leading to strictures and blisters in the mouth and esophagus. This can make eating painful and may lead to dysphagia [29]. A good-quality blender with a large capacity and a high-blend blade speed expressed in rotations per minute can allow families to liquefy healthy fruits and vegetables for the patient to drink. Smoothie drinks can be made fresh and include water or tea (for hydration), cucumbers, greens, bananas, fruit, and protein powders. This allows the person with EB to get optimal nutrition without having to chew or swallow with a tender mouth and esophagus. For optimal nutritional benefit, use fresh fruit rather than juices.

Discussion

Babies born today with EB are living longer, possibly due to a greater understanding of the disease, improved care, and new emphasis on interdisciplinary care plans [5]. This good news means that more babies, children, and even adults are living sometimes for decades with managed EB. The National Epidermolysis Bullosa Registry has collected over 18 years of data from the United States and estimates the incidence of EB at 19.57 per million live births [6]. Rates in other countries vary, sometimes markedly [7]. Since EB respects no borders, it presents itself in the developed and undeveloped world, in cities and rural areas, and with people of all income levels of all races. EB is challenging wherever it occurs, but it can pose conundrums in nations with limited healthcare infrastructure, few healthcare resources, and/or in communities of poverty. While much has been written about EB in peer-reviewed literature, there are few resources that offer basic “tips and tricks” to help manage this condition, knowing that a cure is not yet possible. Thus, it was our goal to offer this article as a singularity in the literature and it is hoped it finds resonance.

Despite the diversity of settings in which EB can occur, there are remarkable similarities. Using semi-structured interviews and qualitative data analysis, one study found that dealing with a child with EB poses a consistent set of challenges, clustering in three main domains: child, family, and caregiver(s) [30]. See Table 1. In the United States, 73% of U.S. families with EB patients reported EB had a “moderate to major” financial impact on the family, and 26% spent over $1,000 per month out of pocket on wound care alone [31]. Other problems, such as lost wages or lost productivity, can be equally devastating but harder to quantify.

**Table 1:** The main concerns in caring for a child with EB in the family are remarkably similar in wealthy or impoverished settings and developed and less-developed countries EB: Epidermolysis bullosa

Domain	Challenges	Comments
EB child	The EB child is “different”	Signs of the disorder can be very visible. Low health literacy of others can cause people to mock or shun the child
	Pain	Few safe and effective long-term analgesic options
	Limitations	Ambulation, activity, and limitations may affect the child’s schooling or education
Family	Uncertainty about condition	Bad days can occur without warning
	Limited employment, recreation	May change or end the employment of one or both parents. Can limit the ability of family members to work, go out, carry on a normal life
	Family stress	Disrupts family routines and may alter relationships, which, in turn, can create feelings of guilt or resentment
	Expenses	Financial stress due to expenses for wound dressings, leading to high out-of-pocket expenses for medical care; expenses for hoists, lifts, and wheelchairs, if needed as well as costs for special transportation
Caregivers	Care-related problems	Changing dressings can take hours every day, not all healthcare providers are knowledgeable about EB so caregivers have limited sources of advice
	Constant need to be available	Always being “on call” can be stressful for even the most dedicated caregiver, who needs to respond to emergencies as they arise
	Lack of skill	Little guidance in care techniques for EB. Few clinicians can offer practical advice, and even the most trusted healthcare professionals may not be good resources (limited experience with EB)
	Resistance to caregiving	The child’s pain and suffering can be emotionally crippling to the caregiver, who may be overwhelmed by a sense of helplessness. Caregivers are not well studied but it may be presumed they are vulnerable to depression and anxiety from this role

The purpose of these “life hacks” for EB children was to create real-world practical tips that have been reported by NoBabyBlisters.org families and may be helpful for caregivers, even those with restricted resources. The goal is to offer some new ideas and tools to a population that is often overlooked by healthcare professionals. These tips came from real-world settings from people caring for others with EB. Advocacy organizations and research scientists are making prolific contributions to finding better treatments and perhaps one day a full-body curative treatment, but simple measures that can be integrated into normal daily life may provide benefit and respite.

This article has several limitations. It is neither a narrative nor a systematic review; there are few such studies in this field. It presents practical tips that are offered as real-world tactics rather than evidence-based guidance.

## Conclusions

EB is a rare, painful, incurable genetic disorder characterized by skin fragility. Few healthcare systems, even from wealthy nations, have resources dedicated to EB patients who can benefit from interdisciplinary care models. Epidemiology data on EB are scarce but the disease occurs all over the world but in such a way that individual patients may be far removed from each other. Researchers, clinicians, and charities have amassed real-world clinical experience with EB, and seven simple tips produced by NoBabyBlisters.org are offered here as a way to provide comfort and relief using simple strategies. People with EB are more comfortable when it is cool when they are protected from ants and other insects, and when they can sit or rest in bean-bag chairs or other furniture that does not shear their fragile skin. These tips may provide some comfort and relief to people with EB.
